# Study find that COVID ARDS was associated with a low risk for possible or proven PCP: still true after dexamethasone use

**DOI:** 10.1186/s13054-022-03886-5

**Published:** 2022-02-10

**Authors:** Patrick M. Honore, Sebastien Redant, Thierry Preseau, Keitiane Kaefer, Leonel Barreto Gutierrez, Sami Anane, Rachid Attou, Andrea Gallerani, David De Bels

**Affiliations:** 1 ICU Department, Centre Hospitalier Universitaire Brugmann-Brugmann University Hospital, ULB University, Place Van Gehuchtenplein, 4, 1020 Brussels, Belgium; 2 ICU Department, Centre Hospitalier Universitaire Brugmann, Brussels, Belgium; 3 ED Department, Centre Hospitalier Universitaire Brugmann, Brussels, Belgium; 4 ICU Department, Centre Hospitalier Universitaire Brugmann, ULB, Brussels, Belgium

**Keywords:** COVID ARDS, PCP, Dexamethasone, High occurrence

Razazi et al. conclude that in their study of patients with COVID acute respiratory distress syndrome (ARDS), they found a low risk for possible or proven *Pneumocystis jirovecii* pneumonia (PCP) [1]. Their findings are in accordance with two smaller studies in France [1] retrieving a low risk of pneumocystis colonization in COVID-19 patients [1]. In their limitation section nothing was said about the fact that none of these COVID patients did receive dexamethasone [1]. We would like to comment as indeed the adjunction of dexamethasone has completely changed the occurrence of COVID-19-associated opportunistic infections including PCP [2]. Coinfection of PCP and COVID-19 has been reported in some patients with COVID-19 till now [2], in which most of them received immunosuppressive therapies, such as corticosteroids [2]. The case reports and reviews are extremely numerous with the use of dexamethasone regarding co-infection of COVID-19 and PCP [2]. Chong et al. reviewed 12 cases of COVID-19 and PCP coinfection [3]. Accordingly, all PCP infections were reported in critically ill patients with COVID-19, which most of them were upon long-term exposure to immunosuppressants (91.7%, 11/12), such as high-dose corticosteroids [3]. Numerous cases were also diagnosed after post-mortem examination [3]. Since PCP is usually reported in patients with T-cell immunodepression, less attention has been paid to PCP in non-immunocompromised intensive care unit (ICU) patients although it accounts for 7% of the co-infections reported in those admitted with Influenza [4]. After the use of dexamethasone in COVID-19 ARDS, these researchers did find an unexpectedly high proportion of ICU COVID-19 patients detected with PCP (10/108 patients; 9.3%) [4]. According to their unexpectedly high proportion of pneumocystis-positive pulmonary samples observed in ICU COVID-19 patients and based on their findings, they advocate systematically searching for pneumocystis in deep respiratory specimens in these patients [4]. They believe that this strategy may be useful in limiting enhanced inflammation due to the presence of pneumocystis in the lung and avoiding inter-patient pneumocystis transmission [4]. It stands to reason that the statement by Razazi et al. that in their study of patients with COVID ARDS, they found a low risk for possible or proven PCP is no longer valid as the systematic use of dexamethasone has increased to an unexpectedly high proportion of ICU COVID-19 patients detected with *P. jirovecii* [4]. It remains somewhat strange that Razazi et al. were not expecting high levels of co-infection of COVID ARDS and PCP after the systematic use of dexamethasone.

## Data Availability

Not applicable.

## Abbreviations

ARDS: Acute respiratory distress syndrome
PCP: *Pneumocystis jirovecii* Pneumonia
ICU: Intensive care unit
