# Supplementary material for: Study find that COVID ARDS was associated with a low risk for possible or proven PCP: still true after dexamethasone use
Source: Crit Care. 2022 Feb 10;26:41. doi: 10.1186/s13054-022-03886-5 (PMC8829981; doi:10.1186/s13054-022-03886-5)
Supplement: Related texts

# Reply: Authors’ response

**Authors:** Romain Arrestier, Armand Mekontso-Dessap, Francoise Botterel, Keyvan Razazi

To the Editor,

We thank Honore and colleagues for their comments on our research letter about low risk of *Pneumocystis* pneumonia in COVID-19 patients with acute respiratory distress syndrome (ARDS).

We point out in the discussion that patients of our cohort were included before the systematic use of dexamethasone, but we may not have sufficiently discussed it.

Enrolment of patients during the first wave is indeed a limitation of our study because of the lower use of corticosteroid therapy compared to the other waves. To explore further this potential bias, we analysed 239 consecutive COVID-19 patients, hospitalized in our intensive care unit during the following waves from 26th May 2020 to 11th April 2021 (*Anticovid study Clinical trials NCT04433105*). All were treated with corticosteroids, 39 (16.3%) with tocilizumab and 29 (12.1%) were immunocompromised.

Of these, 73 had a *Pneumocystis* examination, including 131 PCR and 107 direct examinations (43 May Grunwald Giemsa stainings and 64 Grocott colorations) which were all negative. Only three patients (4%) had a positive PCR with a cycle threshold range of 35.5–37. All were classified as *Pneumocystis* colonization. Two of them did not have any pre-existing risk factors besides 10 days of corticosteroid for COVID-19 and had a negative β-D-glucan. One patient with liver transplantation and long-term corticosteroid therapy and calcineurin inhibitor treatment had a positive PCR with only one low titer positive β-D-glucan (178 pg/mL); he was classified as P*neumocystis* colonization, because his state improved without Trimethoprim-sulfamethoxazole treatment.

These findings are consistent with the finding of Alanio et al. [4] who found 9.3% of *Pneumocystis* positive RT-PCR. We would like to highlight that when discussing *Pneumocystis jirovecii* it is important to differentiate *Pneumocystis* pneumonia and *Pneumocystis* colonization. In the study of Alanio et al., none of the ten patients had *Pneumocystis* pneumonia (PCP) criteria (i.e. positive direct examination for proven PCP or positive RT-PCR with 2 positive β-D-glucan for possible PCP [5]) and were identified as “carriers”. Only 4 of them were treated with prophylactic dose of co-trimoxazole without evident effect on mortality.

All these data are consistent and the statement that *Pneumocystis* pneumonia (PCP) risk is low in COVID-19 ARDS patients is still true in the “era of dexamethasone”, whereas colonization seems to be higher than in the first wave but not associated with mortality. However, further studies are needed to explore if colonized patients have a higher risk of mortality.
